# [Corrigendum] TGF-β1 mediates epithelial to mesenchymal transition via the TGF-β/Smad pathway in squamous cell carcinoma of the head and neck

**DOI:** 10.3892/or.2026.9189

**Published:** 2026-08-31

**Authors:** Changyun Yu, Yong Liu, Donghai Huang, Yaozhang Dai, Gengming Cai, Jinjie Sun, Ting Xu, Yongquan Tian, Xin Zhang

Oncol Rep 25: 1581–1587, 2011; DOI: 10.3892/or.2011.1251

Following the publication of the above paper, it was drawn to the Editor's attention by a concerned reader that, regarding the Transwell assay data shown in Fig. 2C and 4D, two pairs of overlapping sections of data were identified comparing the panels in these figures, where the results from differently performed experiments were intended to have been portrayed.

An Expression of Concern statement was published to account for these concerns (doi: 10.3892/or.2025.9001), after which the authors have responded to the Editorial Office to offer an explanation for this apparent duplication of data within the two figures. To address the issue of the scientific rigor and integrity of this paper, the affected experiments in Figs. 2C and 4D have been performed again in triplicate by the authors. The results obtained were broadly similar to those obtained in the original experiments, and the revised versions of Figs. 2 and 4 are shown on the next page. Also note that the following changes are required to the text in the paper describing the results of these experiments: In the Results section, the corrected data values and text for these experiments should now read as follows: For Fig. 2: ‘The cells that invaded through the pores to the lower surface of the filter were **29±16** and **95±36** (P<0.05), respectively (Fig. 2C and D)’; and for Fig. 4: ‘Smad2 RNAi abrogated the TGF-β1-induced suppression of E-cadherin expression (Fig. 4C) and suppressed the invasion capacity of Tu686 cells (P<0.05, **70±22** vs. **23±8**; Fig. 4D and E)’. The data values have also been amended in the respective figure legends for Figs. 2 and 4, as shown on the next page.

Note that these errors did not have a significant impact on the conclusions reached in this study. The authors regret the errors that were made during the compilation of the original figures, and are grateful to the editor of *Oncology Reports* for allowing them the opportunity to publish this Corrigendum. All the authors agree with the publication of this corrigendum; furthermore, they apologize to the readership for any incon-venience caused.

## Figures and Tables

**Figure 2.**
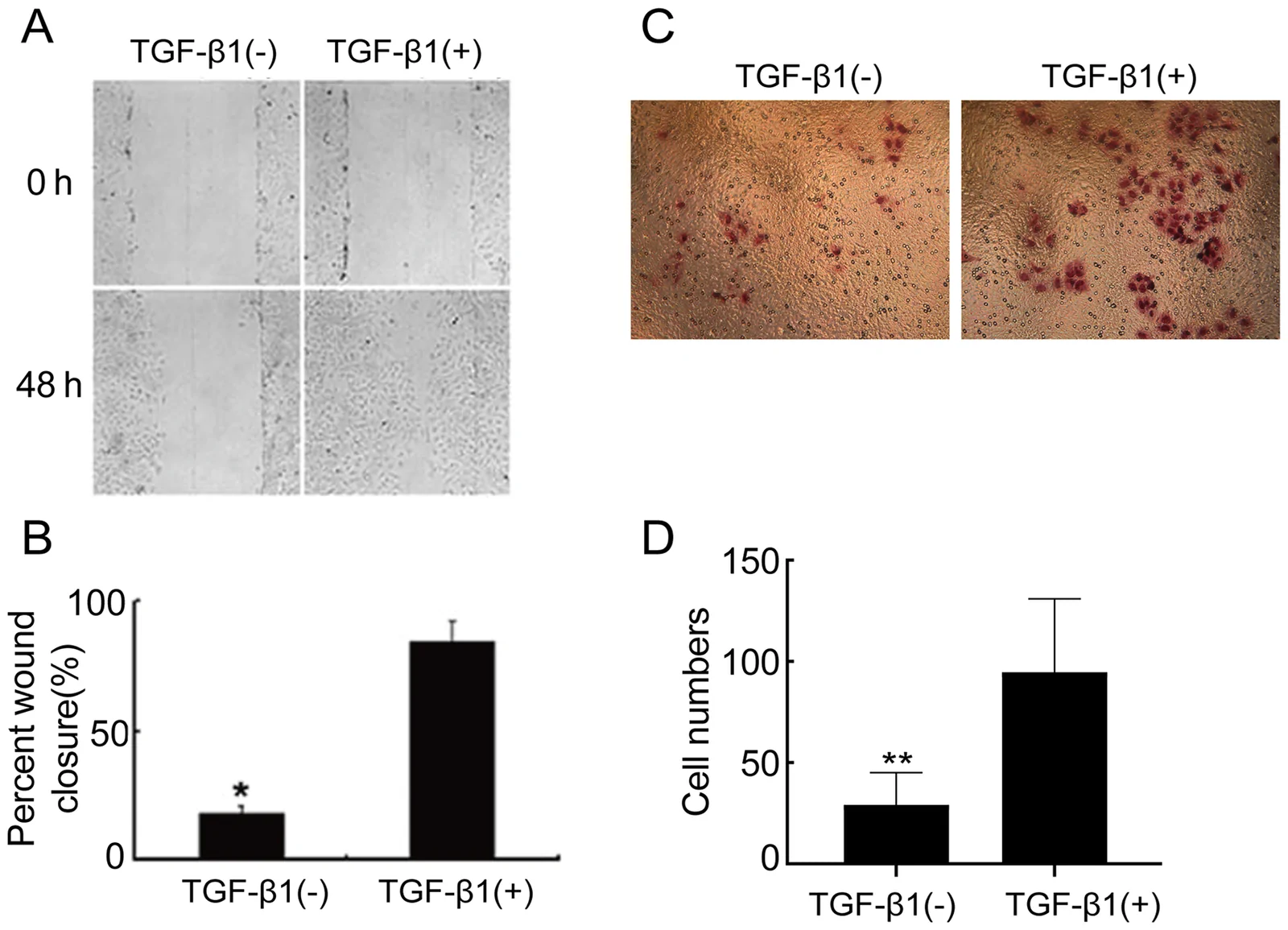
TGF-β1 promoted Tu686 cells migration and invasion (A) The wound healing assay was employed to determine the migration of Tu686 cells. The ‘scratch’ wounds were created by scraping confluent cell monolayer cultured in 6-well plates with a sterile 200 µl pipette tip. After wounding, the cells were cultured with or without 5 ng/ml TGF-β1 for 48 h. Migration of wounded cells was observed and photographed at 0 and 48 h with an inverted Leica phase-contrast microscope (magnification, ×100). (B) The wound healing rate was quantified with measurements of the gap size over time. Closures between untreated and TGF-β1-treated cells at 48 h were 18 and 84% (p<0.05), respectively. (C) The transwell assay was conducted to determine the invasion ability of Tu686 cells. Tu686 cells (2.5×104) were seeded into Matrigel-coated transwells. Cells invaded through chambers in the absence or presence of 5 ng/ml TGF-β1 were photographed and counted. (D) The cells, invaded through the pores to the lower surface of the filter, were counted under a microscope at ×200 magnification. The number of invaded cells was expressed as the average of five random fields. The cells that invaded through the pores to the lower surface of the filter were 29±16 and 95±36 (**P<0.05), respectively. Shown are the representative results of the experiment.

**Figure 4.**
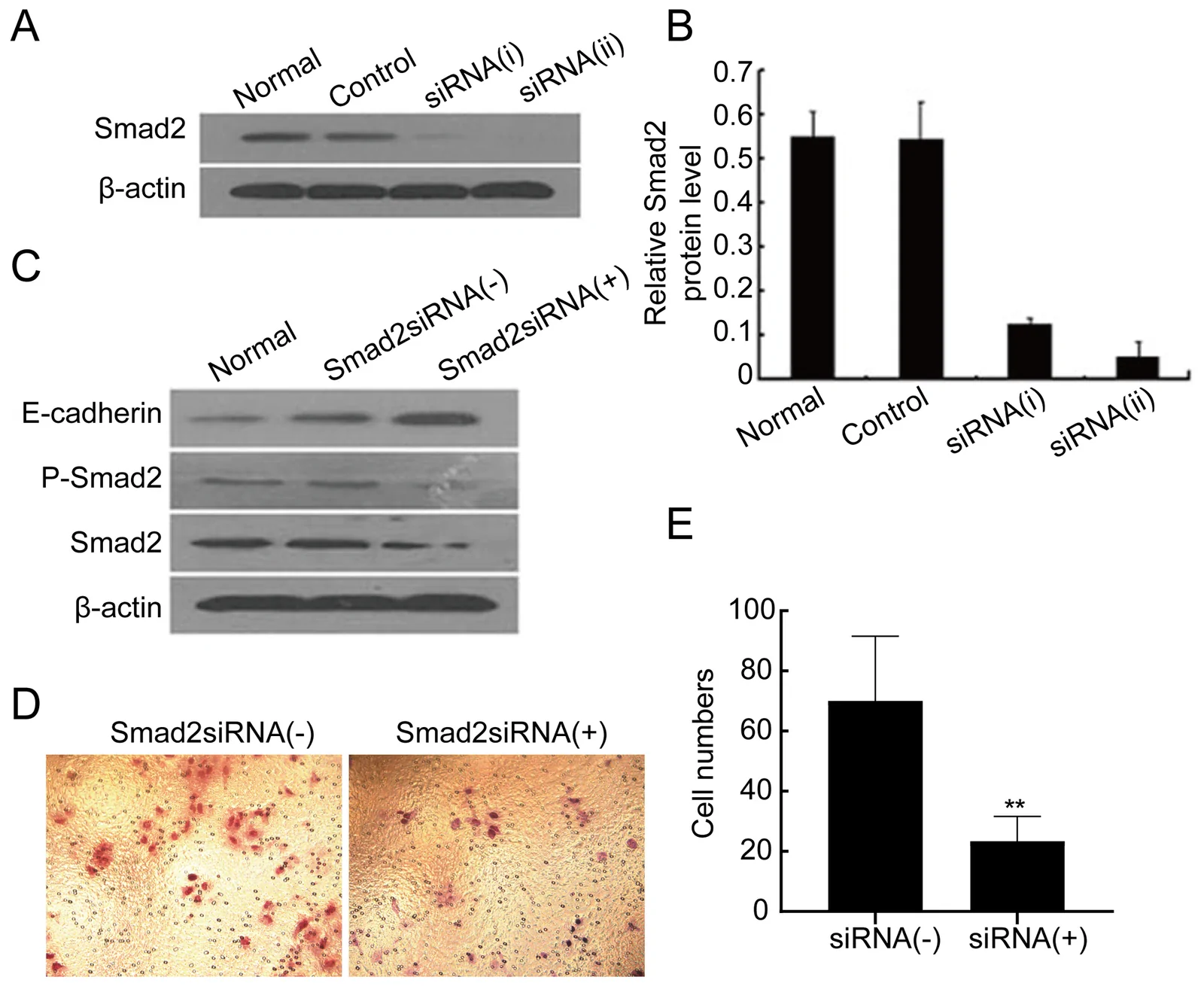
Knockdown of Smad2 by siRNA and its effect on the TGF-β1-mediated changes in Tu686 cells. (A) Western blotting for Smad2 RNAi showing potent silencing of Smad2 with no effect on β-actin (siRNAi, 8 µl siRNA; siRNAii, 10 µl siRNA). (B) expression levels of Smad2 protein in Tu686 cells. As shown, 8 µl siRNA for Smad2 resulted in 78% knockdown, and 10 µl siRNA for Smad2 resulted in a >91% knockdown. (C) Tu686 cells were treated with 5 ng/ml TGF-β1 for 48 h with or without pretreatment with Smad2 RNAi. Then, Western blotting was done for expression of E-cadherin, Smad2, phosphorylated Smad2. E-cadherin expression recovered with low expression of phosphorylated Samd2. (D) For the transwell assay, Tu686 cells, treated with 5 ng/ml TGF-β1 with or without pretreatment with Smad2 RNAi, were seeded into the upper chamber of the transwell for 48 h and the cells that invaded through the pores to the lower surface of the filter were photographed and counted. (E) The cells, invaded through the pores to the lower surface of the filter, were counted under a microscope at ×200 magnification. The number of invaded cells was expressed as the average of five random fields. As shown, invasion capacity of Tu686 cells was suppressed (70±22 vs. 23±8, **P<0.05). Shown are representative results of the experiment.

